# Polyphenols Could Prevent SARS-CoV-2 Infection by Modulating the Expression of miRNAs in the Host Cells

**DOI:** 10.14336/AD.2021.0223

**Published:** 2021-08-01

**Authors:** Dragan Milenkovic, Tatjana Ruskovska, Ana Rodriguez-Mateos, Christian Heiss

**Affiliations:** ^1^Université Clermont Auvergne, INRAE, UNH, F-63000 Clermont-Ferrand, France.; ^2^Department of Internal Medicine, Division of Cardiovascular Medicine, School of Medicine, University of California Davis, Davis, CA 95616, USA.; ^3^Faculty of Medical Sciences, Goce Delcev University, Stip, North Macedonia.; ^4^Department of Clinical and Experimental Medicine, University of Surrey, UK.; ^5^Department of Nutritional Sciences, School of Life Course Sciences, Faculty of Life Science and Medicine, King's College London, London, UK.

**Keywords:** polyphenols, microRNA, miRNA, nutriepigenomic, COVID-19, prevention

## Abstract

Coronaviruses (CoVs) are single-stranded RNA viruses which following virus attachment and entry into the host cell, particularly type 2 pneumocytes but also endothelial cells, release RNA into cytosol where it serves as a matrix for the host translation machinery to produce viral proteins. The viral RNA in cytoplasm can interact with host cell microRNAs which can degrade viral RNA and/or prevent viral replication. As such host cellular miRNAs represent key cellular mediators of antiviral defense. Polyphenols, plant food bioactives, exert antiviral properties, which is partially due to their capacity to modulate the expression of miRNAs. The objective of this work was to assess if polyphenols can play a role in prevention of SARS-CoV-2 associated complications by modulating the expression of host miRNAs. To test this hypothesis, we performed literature search to identify miRNAs that could bind SARS-CoV-2 RNA as well as miRNAs which expression can be modulated by polyphenols in lung, type 2 pneumocytes or endothelial cells. We identified over 600 miRNAs that have capacity to bind viral RNA and 125 miRNAs which expression can be modulated by polyphenols in the cells of interest. We identified that there are 17 miRNAs with both the capacity to bind viral RNA and which expression can be modulated by polyphenols. Some of these miRNAs have been identified as having antiviral properties or can target genes involved in regulation of processes of viral replication, apoptosis or viral infection. Taken together this analysis suggests that polyphenols could modulate expression of miRNAs in alveolar and endothelial cells and exert antiviral capacity.

## Introduction

Faced with the enormous health and socioeconomic burden of the current COVID-19 pandemic, humanity makes every effort to develop effective measures to deal with the crisis in all segments of the society. While social distancing plays an important role, other lifestyle factors, including diet, may have potential to counteract viral susceptibility. In terms of nutrition, in addition to pivotal macro- and micro-nutrients, an adequate and continuous intake of various bioactive compounds, including polyphenols, has been pointed out as a contributing factor in the increased resistance to viral infections [1]. Polyphenols are plant secondary metabolites that have various physiological functions in their dwelling plants. They are widespread *in planta*, with over 8000 different compounds described so far. Many of these compounds are found only in a limited number or in specific plant species [2]. Only about 500 polyphenols are present in the human diet. They are classified as flavonoids, lignans, phenolic acids, stilbenes, non-phenolic metabolites and other polyphenols [3]. Polyphenols have been proven as molecules with strong *in vitro* free radical scavenging capacity. Within the cells however, they act predominantly by modulating the expression of numerous genes [4], including those that are involved in the cellular antioxidant protection [5]. Polyphenols or they metabolites bind to key proteins that are involved in cellular signaling [6, 7], which is one of the mechanisms of their action *in vivo*. They also modulate the level of cellular microRNAs (miRNAs) [8, 9], thus exerting a complex multi-layer regulation of cellular processes.

Experimental studies have shown that polyphenols exert protective effects in respiratory infections caused by various pathogens [10-12], including viruses [13, 14]. The antiviral activity of polyphenols has been investigated *in vitro* and in animal experimental studies. For example, curcumin has been shown to directly inactivate influenza A virus, block its adsorption and inhibit its proliferation *in vitro* [15]. Curcumin increases the survival rate of mice infected with influenza A virus and decreases the production of cytokines by macrophages *in vitro* [16]. These findings pinpoint curcumin as a potentially relevant plant bioactive in prophylaxis of the systemic cytokine storm [17]. Similarly, hesperidin improves influenza A virus (H1N1) induced impairment of pulmonary function in rats by inhibiting cytokine production in pulmonary microvascular endothelial cells [18]. Quercetin [19] and flavanols [20] also exert protective antiviral effects. Although limited, epidemiological studies confirm the protective effect of polyphenols in viral infections [21]. Importantly, recent development of chemically modified epigallocatechin-3-*O*-gallate with improved antiviral efficacy against different types of viruses will significantly contribute to promotion of the use of polyphenols for prevention and treatment of viral infections [22]. A combination of α-glucosyl hesperidin and epigallocatechin gallate was shown to have capacity to induce loss of rotavirus structural integrity as well as reduced infectivity (replication in cell culture) [23]. Given the available data on the protective antiviral actions of polyphenols, there is an urgency to study their potential role in the context of the current COVID-19 pandemic, as prophylactic or therapeutic agents, or as treatment adjunct [24-27]. Quercetin is already included in the protocols for prophylaxis and early outpatient treatment for COVID-19 (https://covid19criticalcare.com). In order to give valuable recommendations for further research on this topic, it is necessary to get a deeper understanding of the interactions between the SARS-CoV-2 virus and the polyphenol induced modulations within the host cells. Therefore, we hypothesize that polyphenols can play an important role in prevention of SARS-CoV-2 associated complications by modulating the expression of host miRNAs that play role in development of the viral infection.

## SARS-CoV-2 and the host cell

Coronaviruses (CoVs) are single-stranded RNA viruses that infect a wide variety of animals, and also humans. In parallel with the efforts for development of effective vaccines for COVID-19, research into the pathogenesis of SARS-CoV-2 infection has also focused on understanding the mechanisms involved in viral entry and replication [28]. Following virus attachment and entry into the host cell, the viral particle is uncoated, and its positive-sense single-stranded RNA genome is released into cytosol where it serves as a matrix for the host translation machinery to produce viral proteins. SARS-CoV-2 RNA encodes four main structural proteins: spike (S), envelope (E), membrane (M), and nucleocapsid (N) and several other accessory proteins, such as 3a, 6, 7a, 7b, 8, and 10 [29]. The spike protein enables the virus to bind to angiotensin-converting enzyme 2 (ACE2) on the host cell membrane, following which the viral genome makes its way inside the host cell [28]. Importantly, the spike protein is the key target for development of SARS-CoV-2 vaccines [30, 31]. Since SARS-CoV-2 utilizes human machinery to translate its RNA after the entry into the cell, it could possibly impact several RNA-binding proteins from the host to bind the viral genome resulting in altered post-transcriptional regulation.

The clinical presentation of COVID-19 varies widely, from asymptomatic infection to a fulminant form with systemic cytokine storm. SARS-CoV-2 infection that is localized mostly in the nose is usually asymptomatic. Progression of the infection to the lower respiratory tract is followed by onset of symptoms and worsening of the clinical presentation [32]. In alveoli, SARS-CoV-2 initially attacks type 2 pneumocytes; subsequently, type 1 pneumocytes are also affected. Although they occupy only a small percentage of the inner alveolar surface, type 2 pneumocytes are present in abundance, and play a critical role in alveolar function by generating the alveolar surfactant layer and acting as progenitor cells for type 1 pneumocytes [33]. Type 2 pneumocytes co-express ACE2, which is the primary cell surface receptor for SARS-CoV-2 and transmembrane serine protease 2 (TMPRSS2), a serine protease that is required to complete the entry process. Co-expression of ACE2 and TMPRSS2 is also found in the nasal secretory cells and absorptive enterocytes, which are also targeted by SARS-CoV-2 [34]. Other receptors and proteins have also been proposed to act as mediators of SARS-CoV-2 entry in the human cells, such as sialic acid receptor, CD147, or cathepsin B and L. All these molecules are also expressed in the endothelial cells [35], suggesting their involvement in the major vascular derangements in COVID-19. Indeed, lung autopsy specimens of patients who died from respiratory failure caused by SARS-CoV-2 show severe endothelial damage with disruption of endothelial membranes and presence of SARS-CoV-2 within the endothelial cells, which is accompanied with widespread vascular thrombosis, microangiopathy and occlusion of alveolar capillaries [36]. Infection of endothelial cells and endotheliitis in COVID-19, however, are not limited to the lungs, and have been demonstrated in different organs, such as kidney, liver, heart, or small intestine [37]. Therefore, endothelial and alveolar cells are among the primary targets in developing of strategies to combat SARS-CoV-2, including studies on polyphenols as potentially protective factors.

## Interplay between host cell miRNAs and viral RNAs

Replication cycle of SARS-CoV-2 potentially exposes its RNA to an antiviral cellular defense that relies on the host’s endogenous miRNAs. These miRNAs could directly degrade viral RNA and/or prevent viral protein translation, such as has been reported for the influenza virus [38]. The role of host cellular miRNAs in negative regulation of virus replication is not surprising because siRNAs, which inhibit mRNA expression by comparable mechanism, have been shown to effectively impair virus replication. The first evidence that host cells might employ a miRNA in antiviral defense was provided by Lecellier et al., showing that human miR-32 restricts the accumulation of primate foamy virus type 1 (PFV-1) in human cells by targeting viral RNA transcripts [39]. Similarly, miR-24 and miR-93 can target vesicular stomatitis virus (VSV) L and P proteins [40] and miR-29a targets human immunodeficiency virus (HIV) Nef proteins [41] to inhibit replication. Therefore, the use of antisense oligonucleotides to block viral genome can be the most straightforward and easy to implement way towards development of new antiviral therapies. For example, in one study researchers developed four artificial miRNAs that were designed to target different regions of Chikungunya virus (CHIKV) genome. These miRNAs significantly inhibited CHIKV replication, up to 99.8% [42]. On the other hand, miR-122 upregulates the replication of the HCV RNA genome, having a key role in promoting viral RNA stability [43]. The miRNA-based therapy for HCV infection is performed using an antimiR which was designed to bind directly to the mature strand of the targeted miR-122 and thus to induce a functional blockade. This is a great example of therapeutic miRNA targeting with a significant reduction in HCV titers (> 300-fold), and without signs of a rebound even after discontinuation of the treatment [44]. Viral RNAs can also serve as miRNA sponges and reduce cellular miRNA levels, which in consequence can modulate the host’s cellular processes to facilitate viral replication or to inhibit the host’s antiviral responses. Regarding SARS-CoV-2, it has been proposed that bats, considered as its host of origin, have tolerance to potentially deadly viruses because of the specific miRNA profile [45].

## miRNAs could explain diversity in response to SARS-CoV-2 infection

Numerous reports have illustrated the large diversity in terms of responses and clinical outcomes to SARS-CoV-2 infection. One possible variable that has not been sufficiently addressed are the individual differences in patients’ miRNA profiles. A recent study has indeed suggested that SARS-CoV-2 virulence in aged patients may be due to a lower abundance of miRNAs, which may be a contributing factor in disease severity [46]. Moreover, in the COVID-19 pandemic, a significant gender disparity was evidenced with the male lethality higher than the female one: in Italy 17.7% vs 10.7% [47]. Sex chromosomes, in particular X chromosome, and sex hormones are key actors in these differences. Interestingly, a different expression in males and females of several miRNAs has been observed owing to sex hormones differences and/or localization on the X-chromosome, which is particularly enriched for miRNAs. Hence, the potential role of these gender-associated miRNAs in immunity regulation and in modulation of viral receptors and co-receptors should be considered as a crucial factor in the observed different pathogenicity and lethality of COVID-19 in men and women. These observations suggest that the individual epigenetic differences in the miRNA profiles during an infection could affect the effectiveness of the antiviral responses and the disease severity. Thus, modulating or manipulating endogenous miRNAs to improve immunity and clinical outcomes in SARS-CoV-2 infection could be another avenue for further investigation [47].

## Identification of human miRNAs that could interact with SARS-CoV-2

Bartoszewski et al. [48] examined whether SARS-CoV-2 could be targeted by human endogenous miRNAs. The coronaviruses genomes were tested against the set of 896 confident mature human miRNA sequences that were obtained from the miRBase v2.21 using the RNA22 v2 microRNA target discovery tool web server [49]. The authors observed that the pathogenic human coronaviruses could interact with host-specific miRNAs, disturb gene expression and consequently suppress immunity or prevent activation of unfolded protein response (UPR)-related apoptosis. The authors focused on the potential role of the 28 miRNAs that were uniquely specific for SARS-CoV-2. The majority of these miRNAs are well expressed in bronchial epithelial cells, and their dysregulation has been reported for various human lung pathologies that include chronic obstructive pulmonary disease, cystic fibrosis, and tuberculosis. Furthermore, many of these miRNAs have been proposed to act as tumor suppressors that target apoptosis-related pathways. Hence, SARS-CoV-2, by its potential to reduce host’s miRNA pool, may promote infected cell survival and thus continuity of its replication cycle. The authors concluded that the pathogenesis of human coronavirus could involve modulation of host miRNA levels by acting as miRNA sponges to facilitate viral replication and/or to avoid immune responses.

Sacar Demirci and Adan [50] performed a machine learning based miRNA prediction analysis for the SARS-CoV-2 genome and identified human miRNAs that appeared to be able to target viral genes encoding proteins that are involved in viral life cycle. Among 2,654 mature entries of Homo sapiens in miRBase, 479 of them could target SARS-CoV-2 genes. The SARS-CoV-2 genes targeted by host-cellular miRNAs are mainly responsible for viral biogenesis, entrance, replication and infection. For instance, miR-203b-3p can target ORF1ab and ORF3a which result in the suppression of viral replication as already shown in influenza A virus [51]. let-7c-5p can target ORF1ab in SARS-CoV-2 while it was found to be involved in H1N1 influenza A suppression by targeting its M1 protein [52]. The presence of such miRNAs could be considered as a host’s innate antiviral defense mechanism.

Srivastava *et al*. [53] used computational approach to investigate the potential binding sites of human miRNAs in SARS-CoV-2 genome using the Find Individual Motif Occurrences (FIMO), a motif-based sequence analysis tool [54]. They identified 22 miRNAs that could potentially bind throughout the length of the SARS-CoV-2 viral genome. These results suggest that several important miRNAs are likely being titrated by SARS-CoV-2 genome that could result in dysregulation of post-transcriptional networks in the infected cells. Majority of the identified miRNAs were highly expressed in immune cells including CD8^+^T cells, CD4^+^T cells, NK cells, CD^+^14 cells and mast cells, signifying that these miRNAs could contribute to the progression of the viral infection and host immune response. The results also indicate that the highly confident genes targeted by these sponged miRNAs were enriched for functional terms including ‘post transcriptional gene regulation’ and ‘cell to cell communication’ suggestive of a large-scale dysregulation across host’s tissues.

Guterras et al. [55] analyzed sixty SARS-CoV-2 genomes to identify regions that could work as miRNA sponges and potentially bind human miRNA. They searched for miRNAs that shared 100% identity of the 8mer seed region with SARS-CoV-2 genome regions, both positive- and negative-sense. Among 2.654 mature miRNA sequences in miRBase, they identified over 1000 miRNAs that showed 100% of similarity between their sequences and viral RNA. The search for miRNAs was then refined to identify those that interact with the SARS-CoV-2 genome with a perfect alignment of 11 nucleotides encompassing the seed region. Using this approach, 34 miRNAs for positive-sense and 45 miRNAs for negative-sense viral RNA were identified that can strongly bind to certain key SARS-CoV-2 genes. Literature review revealed the potential of these miRNAs in the immunopathogenesis and potential treatment possibilities of COVID-19. The disruption and dysfunction of these miRNAs may dysregulate the immune response and stimulate the release of inflammatory cytokines altering the cellular response to viral infection. These miRNAs have been identified in studies related to pulmonary and cardiac disorders, including lung cancer, asthma, pneumonia, or cardiac fibrosis, among others.

Khan *et al*. [56] also used bioinformatic analysis pipeline which allowed them to identify 106 host antiviral miRNAs against SARS-CoV-2. Whilst comparing these miRNAs with the antiviral miRNAs from VIRmiRNA [57], they have found three (miR-17-5p, miR-20b-5p, and miR-323a-5p) host miRNAs against SARS-CoV-2 which have experimental evidence of having antiviral roles during infections. Next, they performed miRNA pathway enrichment analysis and found that several pathways might be modulated by the host miRNAs to suppress the entry of the virus, prevent the spread of the virions, and to minimize the systemic symptoms resulting from the infection. For example, host miRNAs might have a probable role in blocking the entry of the virus, as they are found to be targeting the pathways needed for viral entry, such as PDGF receptor-like signaling, Arf-6 signaling, PI3K-Akt signaling, EGFR signaling, signaling evens mediated by focal adhesion kinase, CDC42 signaling, the EphrinB-EPHB pathway, Cadherin signaling, or RTK signaling. They can also block some machinery like p38 MAPK signaling, FAK signaling, or PI3K-Akt signaling, which can be hijacked by viruses for their efficient replication, pre-mRNA processing, and translation. These host miRNAs might also try to reduce some host-induced inflammatory responses to prevent acute lung damage by targeting pathways such as VEGF signaling, Integrin signaling, TGF-beta signaling or TRAIL signaling. Some signaling pathways, such as CXCR4 signaling, TGF-beta signaling, mTOR signaling, or PI3K-Akt signaling, can facilitate viral survival in infected cells by inhibiting apoptosis, autophagy, or early immune responses.

Therefore, to develop effective therapeutics, there is a need to decipher the cellular targets in humans that interact with SARS-CoV-2 and result in altered functional outcomes. All these bioinformatic analyses discovered that human miRNAs harbor binding sites across the SARS-CoV-2 genome that could act as post-transcriptional regulators. Therefore, miRNA regulation or manipulation could provide a novel basis for antiviral therapies. We and others have shown that miRNA expression profiles are often cell-type specific and differ between individuals, and importantly, can be affected by different factors such as nutrients present in our diets, including polyphenols.

## Antiviral properties of polyphenols

Together with well described properties of polyphenols to prevent or delay development of noncommunicable diseases such as cardiovascular, metabolic or neurodegenerative disorders, studies, both *in vitro* and *in vivo*, have also revealed their antiviral properties either by entering into the defensive mechanism directly through interfering with the target viruses, or indirectly through activating the cells associated with the adaptive immune system [58]. Several studies have described potential antiviral activity of plant food bioactives against herpes virus, human immunodeficiency virus, influenza virus or hepatitis C virus [59]. In a recent review, it can be observed that different polyphenols, such as quercetin, quercetin 3-glucoside, resveratrol, rutin, kaempferol, catechin, epigallocatechin, epigallocatechin gallate, or ferulic acid derivatives can inhibit the replication of both influenza A and B viruses, suppress the influenza virus-induced cell death, or inhibit viral activity in the early stage of flu infection [60]. These effects have been associated with the capacity of polyphenols to exert anti-inflammatory and immunomodulatory effects (such as modulation of the expression of cytokines including IL-6, IL-8 and IFN-β) or capacity to modulate molecular pathways associated with viral infection (such as Toll-like receptor 7 signaling pathway, cell cycle pathway or autophagy related pathways). Several studies have also observed potential anti-Zika virus activity of polyphenols, such as the citrus flavanones [61, 62], flavonoids [63] or quercetin-3-β-*O*-D-glucoside [64]. Antiviral activity against Ebola virus has been reported for several polyphenols, such as quercetin [65], quercetin 3-β-*O*-D-glucoside [66] or epigallocatechin-3-gallate [67]. Moreover, several studies have revealed potential antiviral effect of polyphenols against other coronaviruses such as Middle East respiratory syndrome-coronavirus (MERS-CoV). For example, experimental and computational study showed that flavonoids (herbacetin, isobavachalcone, quercetin 3-β-D-glucoside and helichrysetin) can block the enzymatic activity of MERS-CoV 3CL protease which regulates replication of the MERS-CoV [68].

**Table 1:** Polyphenol-modulated miRNAs in pneumocytes, lung and endothelial cells.

Cells/Tissue	Polyphenol	Concentration	Significantly modulated miRNAs	Ref
A549 cells	Curcumin	10 - 20 μM	miR-206	76
A549 cells	Curcumin	10 μM	hsa-miR-330-5p; hsa-miR-331-5p; hsa-miR-1276; hsa-miR-544a; hsa-miR-29c-5p; hsa-miR-335-5p; hsa-miR-296-3p; hsa-miR-34a-5p; hsa-miR-26a-1-3p; hsa-miR-190a; hsa-miR-362-3p; hsa-let-7f-2-3p; hsa-miR-302b-3p; hsa-miR-338-3p; hsa-miR-455-3p; hsa-miR-29c-3p; hsa-miR-154-3p; hsa-miR-21-3p; hsa-miR-377-5p; hsa-miR-34c-5p; hsa-miR-1257; hsa-miR-744-3p; hsa-miR-502-5p; hsa-miR-33a-3p; hsa-miR-424-3p; hsa-miR-92a-1-5p; hsa-miR-10b-3p; hsa-miR-769-3p; hsa-miR-1179; hsa-miR-516a-3p; hsa-miR-148a-5p; hsa-miR-604; hsa-miR-499a-5p; hsa-miR-1262; hsa-let-7a-3p; hsa-miR-25-5p	77
A549 cells	Curcumin	50 - 100 μM	miR-98	78
A549 cells	Curcumin	20 - 40 μM	miR-21	79
A549 cells	Curcumin	15 μM	miR-320; miR-26a; let-7i; miR-130a; miR-16; miR-125b; miR-23a; miR-27b; miR-155; miR-625; miR-576-3p; miR-186*; miR-9*; let-7e	80
Rat lung	Quercetin	Intra-peritoneal daily injections of 30 mg/kg Quercetin for 3 weeks.	miR-204	71
Human microvascular endothelial cells (HMEC-1)	Curcumin	1 - 5 μM	miR-126	81
HUVECs	Resveratrol	50 μM	miR-221; miR-222	82
HUVECs	Curcumin	10 μM	miR-93	83
HUVECs	Purified epicatechin metabolites: 3′-O-methyl(-)-epicatechin (3′MEC), 4′-O-methyl(-)-epicatechin-7-β-D-glucuronide (4′MEC7G) and (-)-epicatechin-4′-sulfate (EC4′S)	Mixture of 3′MEC, 4′MEC7G and EC4′S, 1 μM each	hsa-let-7a; hsa-let-7f; hsa-miR-10a; hsa-miR-10b; hsa-miR-1290; hsa-miR-130b; hsa-miR-134; hsa-miR-181a*; hsa-miR-221*; hsa-miR-224; hsa-miR-30a*; hsa-miR-30c; hsa-miR-30e*; hsa-miR-320a; hsa-miR-320c; hsa-miR-320d; hsa-miR-361-5p; hsa-miR-365; hsa-miR-543; hsa-miR-769-5p	84
HUVECs	Mixture A or/and Mixture B	One mixture contained compounds present in the circulation 1-5 h after consumption of anthocyanin-rich sources, named mix A. It was composed of cyanidin-3-arabinoside, cyanidin-3-galactoside, cyanidin-3-glucoside, delphinidin-3-glucoside, peonidin-3-glucoside and 4-hydroxybenzaldehyde. Once dissolved in the culture medium, the final concentrations were 0.1 μM for anthocyanins and 0.5 μM for 4-hydroxybenzaldehyde. The other mixture contained metabolites present in the circulation for around 15 h after the consumption, named mix B. It was composed of hippuric acid, vanillic acid, ferulic acid and protocatechuic acid at final concentration in the culture medium of 2 μM, 2 μM, 1 μM and 0.2 μM, respectively.	Mix A/(+) TNF: hsa-let-7f; hsa-miR-126*; hsa-miR-1260; hsa-miR-1268; hsa-miR-130a; hsa-miR-181b; hsa-miR-1915; hsa-miR-26a; hsa-miR-30b; hsa-miR-361-5p; hsa-miR-374a; hsa-miR-376c; hsa-miR-455-3p; hsa-miR-99b Mix B/(+) TNF: hsa-let-7a; hsa-let-7f; hsa-miR-1246; hsa-miR-125b; hsa-miR-126*; hsa-miR-1260; hsa-miR-1275; hsa-miR-196b; hsa-miR-216a; hsa-miR-23a; hsa-miR-374a; hsa-miR-720; hsa-miR-923 Mix A+B/(+) TNF: hsa-let-7c; hsa-let-7f; hsa-miR-1207-5p; hsa-miR-1246; hsa-miR-125a-5p; hsa-miR-125b; hsa-miR-1305; hsa-miR-181b; hsa-miR-1915; hsa-miR-361-5p; hsa-miR-572; hsa-miR-575; hsa-miR-638	6
HUVECs	Curcumin	40 μM	hsa-miR-4298; hsa-miR-23a; hsa-miR-423-3p; hsa-miR-671-5p; hsa-miR-21; hsa-miR-29b; hsa-miR-27a; hsa-miR-491-5p; hsa-miR-1307; hsa-miR-181a; hsa-miR-92a; hsa-miR-339-5p; hsa-miR-25; hsa-miR-27b; hsa-miR-941; hsa-miR-222; hsa-miR-138; hsa-miR-149; hsa-miR-1226; hsa-miR-3198; hsa-miR-1973; hsa-miR-665; hsa-miR-3127; hsa-miR-196b; hsa-miR-30e; hsa-miR-1246; hsa-let-7g; hsa-miR-1979; hsa-miR-1275; hsa-miR-1975	85
Human microvascular endothelial cells (HMEC-1)	Quercetin	30 μM	miR-216a	86
Human retinal endothelial cells (HRECs)	Resveratrol	50 μM	miR-15a	87
HUVECs and HRECs	HT-3O sulfate (HT-3Os), the major plasma metabolite of Hydroxytyrosol (HT)	10 μM	let-7	88
HUVECs (CRL-1730)	Resveratrol	10 - 50 μM	miR-126	89

## Polyphenols and miRNA expression in alveolar and endothelial cells

Polyphenols exert beneficial effects on lung health, which has been demonstrated in several animal models. For example, prophylactic resveratrol inhalation partially preserves lung function in prematurely aging mice [69], whereas oral treatment with curcumin and resveratrol, individually or in combination, has beneficial effects in lung carcinogenesis [70]. It has also been demonstrated that quercetin can ameliorate lung inflammation in pulmonary artery hypertension, which is mediated by miR-204 modulation [71]. Interestingly, *in vitro* studies demonstrating beneficial effects of polyphenols in lung function, were performed mostly on type 2 pneumocytes. In the majority of these studies the A549 cell line was used, a human lung adenocarcinoma cell line that is subsequently characterized as a representative to type 2 pneumocytes [72]. Using this cell line, for example, the anti-inflammatory and antioxidant effects of polyphenols have been demonstrated [73, 74], as well as their involvement in reducing the toxicity of PM 2.5 particles [75].

**Figure 1.**
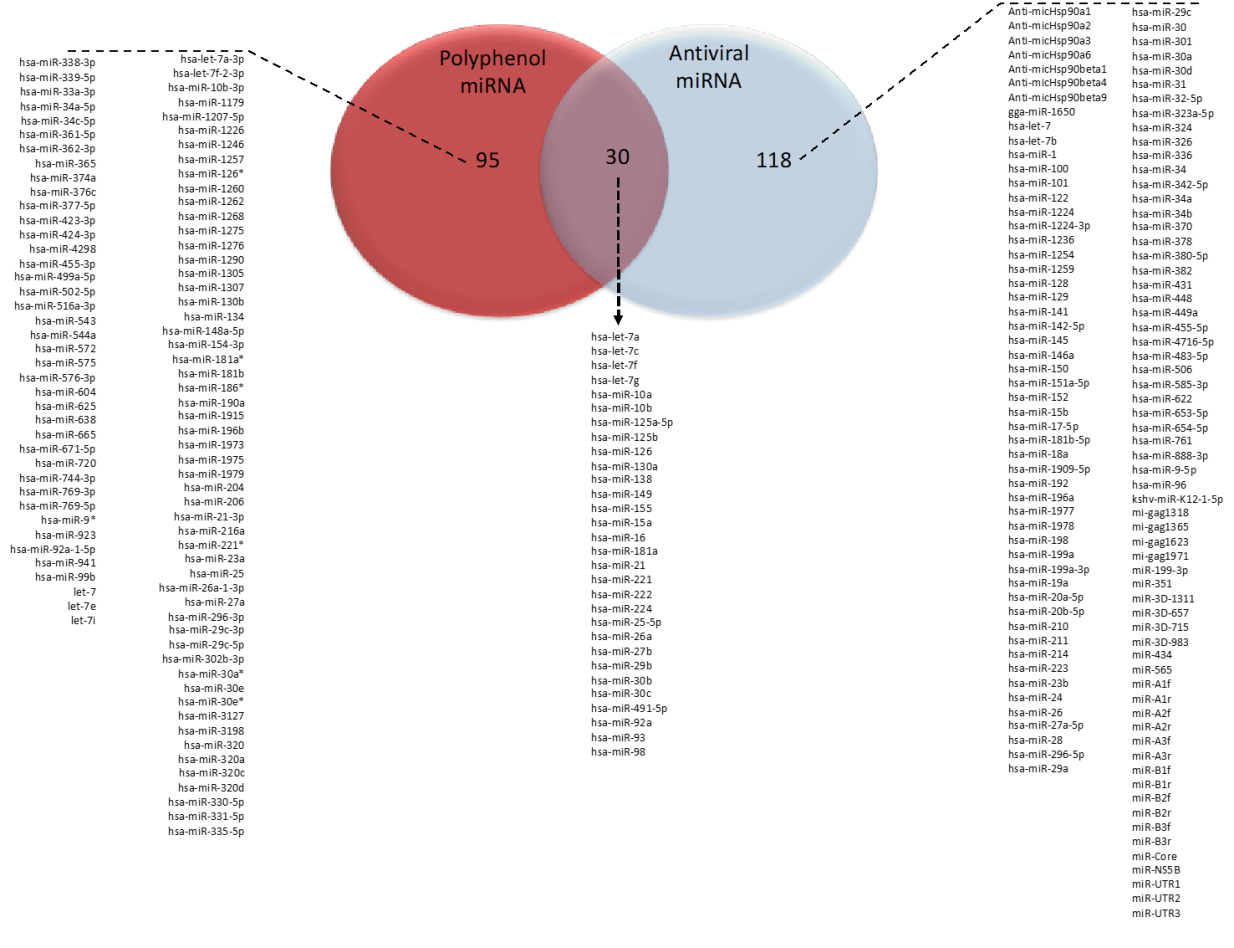
Venn diagram showing the overlapping between microRNAs with antiviral properties and microRNAs which expression can be modulated by polyphenols in type 2 pneumocytes and endothelial cells.

Even though studies demonstrated that polyphenols exert their health properties through modulation of expression of protein-coding genes and proteins, there are only few of them that investigated the effects of polyphenols on the modulation of miRNAs in type 2 pneumocytes. We were able to identify a total of five such studies [76-80], where the expression of miRNAs was analyzed by RT-PCR or microarray methods. The significantly modulated miRNAs are extracted in the Table 1. On the other hand, the number of *in vitro* studies on polyphenol-induced miRNA modification in endothelial cells is higher, and we were able to identify a total of ten such studies (Table 1), where the expression of miRNAs was also analyzed by RT-PCR or microarray methods [6, 81-89]. The most commonly used model of endothelial cells are human umbilical vein endothelial cells (HUVECs), but other models of endothelial cells are also used such as human microvascular endothelial cells (HMEC-1 cell line) [81, 86, 90], or human retinal endothelial cells (HRECs) [87, 88]. These experimental studies have demonstrated beneficial effects of polyphenols on the endothelial function, in the first place in the context of the complex pathophysiology of atherosclerosis, but diabetic retinopathy has also been studied. Still, given the findings from the scanning electron microscopy of lung tissue obtained during autopsy of patients who died from COVID-19, where the presence of the virus in the endothelial cells is clearly visible [36], the above experimental data represent a valuable source of information that will help us to get a deeper insight into potential effects of dietary polyphenols on SARS-CoV-2 exposure.

These studies have identified 125 different miRNAs differentially expressed by polyphenols in alveolar or endothelial cells. Few of the miRNAs have been identified in more than 1 study, for example, modulation of expression of hsa-let-7f and hsa-miR-126 have been observed in 4 different studies, hsa-miR-1246, hsa-miR-125b, hsa-miR-23a and hsa-miR-361-5p in 3 studies. Moreover, among these miRNAs, some have been identified as presenting antiviral activities. We used VIRmiRNA [57] to obtain the list of miRNAs with antiviral properties, that included 148 different miRNAs. Comparison with 125 miRNAs identified as modulated by polyphenols in alveolar and endothelial cells, has demonstrated that 30 are in common (hsa-let-7a, hsa-let-7c, hsa-let-7f, hsa-let-7g, hsa-miR-10a, hsa-miR-10b, hsa-miR-125a-5p, hsa-miR-125b, hsa-miR-126, hsa-miR-130a, hsa-miR-138, hsa-miR-149, hsa-miR-155, hsa-miR-15a, hsa-miR-16, hsa-miR-181a, hsa-miR-21, hsa-miR-221, hsa-miR-222, hsa-miR-224, hsa-miR-25-5p, hsa-miR-26a, hsa-miR-27b, hsa-miR-29b, hsa-miR-30b, hsa-miR-30c, hsa-miR-491-5p, hsa-miR-92a, hsa-miR-93, hsa-miR-98) (Fig. 1). Among these common miRNAs is miR-221 which has been identified as presenting antiviral ability against respiratory syncytial virus in bronchial epithelial cells [91]. Regarding hsa-miR-125b, its antiviral activities were described for human papillomavirus and respiratory syndrome [92, 93]. Taken together this analysis suggests that polyphenols could modulate expression of miRNAs in alveolar and endothelial cells and exert antiviral capacity.

## Modulation of miRNAs by polyphenols related to SARS-CoV-2

Our next step was to compare the list of human miRNAs that may interact with SARS-CoV-2 genome with the miRNAs which expression can be affected by polyphenols in alveolar and/or endothelial cells. We identified 644 miRNAs from the literature that can interact with SARS-CoV-2 genome (based on the publications of Bartoszewski *et al*. [48], Sacar Demirci and Adan [50], Srivastava *et al*. [53], Guterres *et al*. [55], and Khan *et al*. [56]) and 125 miRNAs which expression can be modulated by polyphenols. Comparison of the 2 lists revealed 17 miRNAs in common: hsa-let-7a-3p, hsa-miR-25-5p, hsa-miR-1246, hsa-miR-125a-5p, hsa-miR-1262, hsa-miR-1290, hsa-miR-148a-5p, hsa-miR-154-3p, hsa-miR-21-3p, hsa-miR-320c, hsa-miR-335-5p, hsa-miR-34a-5p, hsa-miR-377-5p, hsa-miR-455-3p, hsa-miR-499a-5p, hsa-miR-544a and hsa-miR-744-3p. This analysis suggests that polyphenols could modulate miRNA expression of host cells and potentially improve outcome upon SARS-CoV-2 exposure.

Among the miRNAs in common is hsa-miR-1246. It has been observed that this miRNA has homologous sequence to *ACE2*, which plays role in integration with the virus and mediates viral entry. One recent study reported higher expression of ACE2 in the small airway epithelium of smokers compared to nonsmokers [94]. The same study observed that the expression of miR-1246 is downregulated in the cells of smokers compared with nonsmokers, suggesting that miR-1246 plays a role in the regulation of ACE2, thus shedding light at the pathogenesis of COVID-19 and risk factors in the population. On the other hand, it was reported that curcumin can increase the expression of this miRNAs in endothelial cells [85]. This could suggest that consumption of curcumin would increase the expression of hsa-miR-1246, lower ACE2 levels and consequently the SARS-CoV-2 infection. It was also reported that human alveolar basal epithelial cells infected with influenza A viruses can modulate the expression of 20 miRNA, including up-regulation of miR-1290 [95]. When miR-1290 antagonist, LNA-1290 is administrated to the cells, there is significant reduction of viral protein levels and viral titers [96]. Interestingly, we have described that flavanols can decrease the expression of this miRNA in endothelial cells [84]. Several studies also suggested the role of miR-21-3p in viral infection. For example, in A549 cells, miR-21-3p promoted replication of influenza A virus [97]. Infection of A549 cells with influenza A virus (H1N1 or H5N1) in the presence of miR-21-3p inhibitor significantly decreased the viral load in the cells probably through regulation of HDAC8. On the other hand, in A549 cells exposed to curcumin, a significant down-regulation in the expression of this gene has been identified [77]. Potential role in viral and/or antiviral properties have been described for few other common miRNAs, such as let-7a-3p [98], miR-125a-5p [99] or miR-455-3p [100].

**Figure 2.**
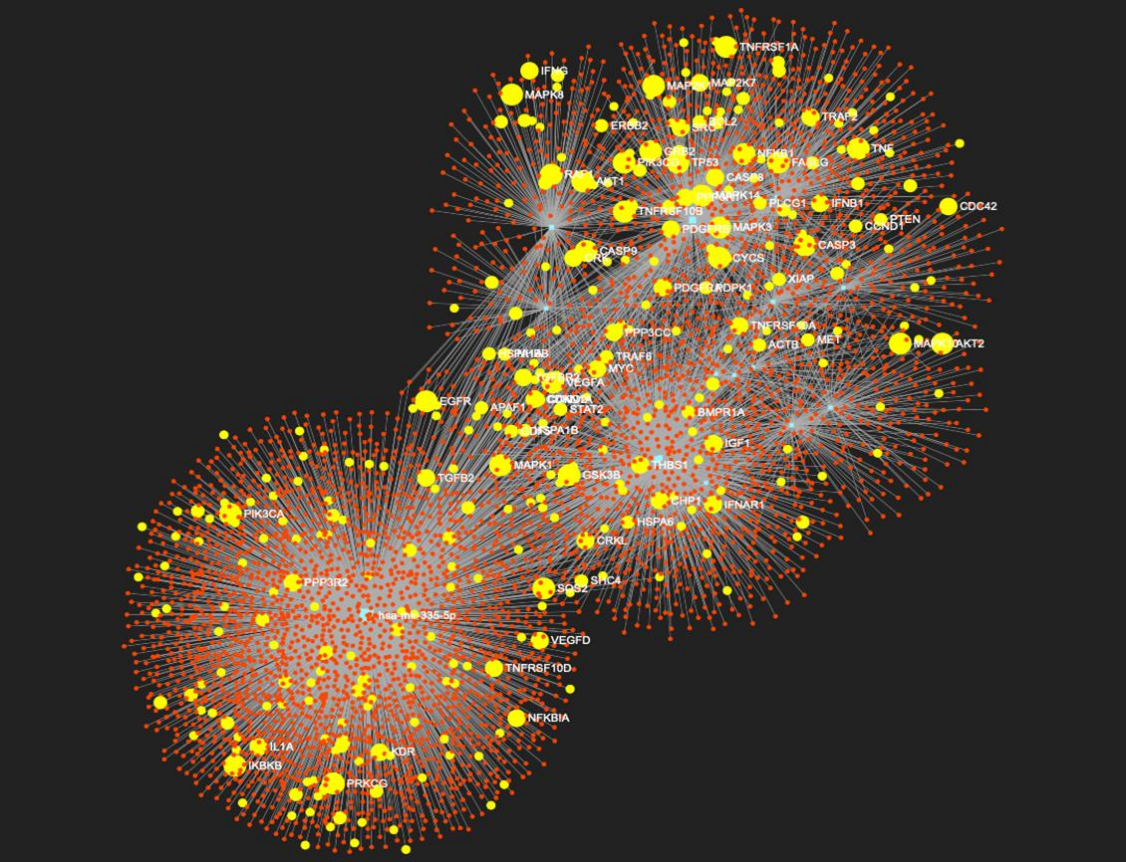
Network between 17 miRNAs, common between cellular miRNAs than can interact with SARS-CoV-2 RNA and miRNAs modulated by polyphenols, and their potential target genes. Blue circles represent miRNA, red circles are their potential targets. In yellow are target genes involved in pathways regulating virus entry, replication and viral infections.

Together with literature analysis, we also performed bioinformatic analysis to identify potential target genes of these 17 common miRNAs. Using MirWalk database (http://mirwalk.umm.uni-heidelberg.de) [101] we identified 1734 potential targets with minimum free energy <-20kcal/mol indicating a stronger predicted pairing between the miRNA and the target mRNA. With the aim to identify potential pathways in which these target genes are involved in we performed pathway analysis using MirWalk database as well as GeneTrail2 database. Interestingly, both databases showed that the potential target genes are involved in different cellular pathways, including those that have been previously identified as involved in regulation of SARS-CoV-2 replication [56]. These pathways include EGFR signaling, focal adhesion kinase, integrin signaling, mTOR signaling, p38 MAPK signaling, PDGF receptor-like signaling, PI3K-Akt signaling and VEGF signaling, described to regulate entry of the virus, its efficient replication, inflammatory responses or viral survival in infected cells (Fig. 2). Moreover, comparison of the target genes with genomic data present in databases using Enrichr tool (https://maayanlab.cloud/Enrichr/) [102] revealed significant homology with gene expression of healthy lung biopsy vs. SARS-CoV-2 infected lung, SARS-CoV-2 infection in lung tissue or A549 infected with SARS-CoV-2. Therefore, from the data on the effect of polyphenols on expression of miRNAs which potentially can target the SARS-CoV-2 genome as well as their potential targets, it could be suggested that these bioactives modulate expression of miRNAs in the host cells in such a way to prevent viral entry, its replication and survival, thus preventing or lowering the risk of development of complications in COVID-19.

**Figure 3.**
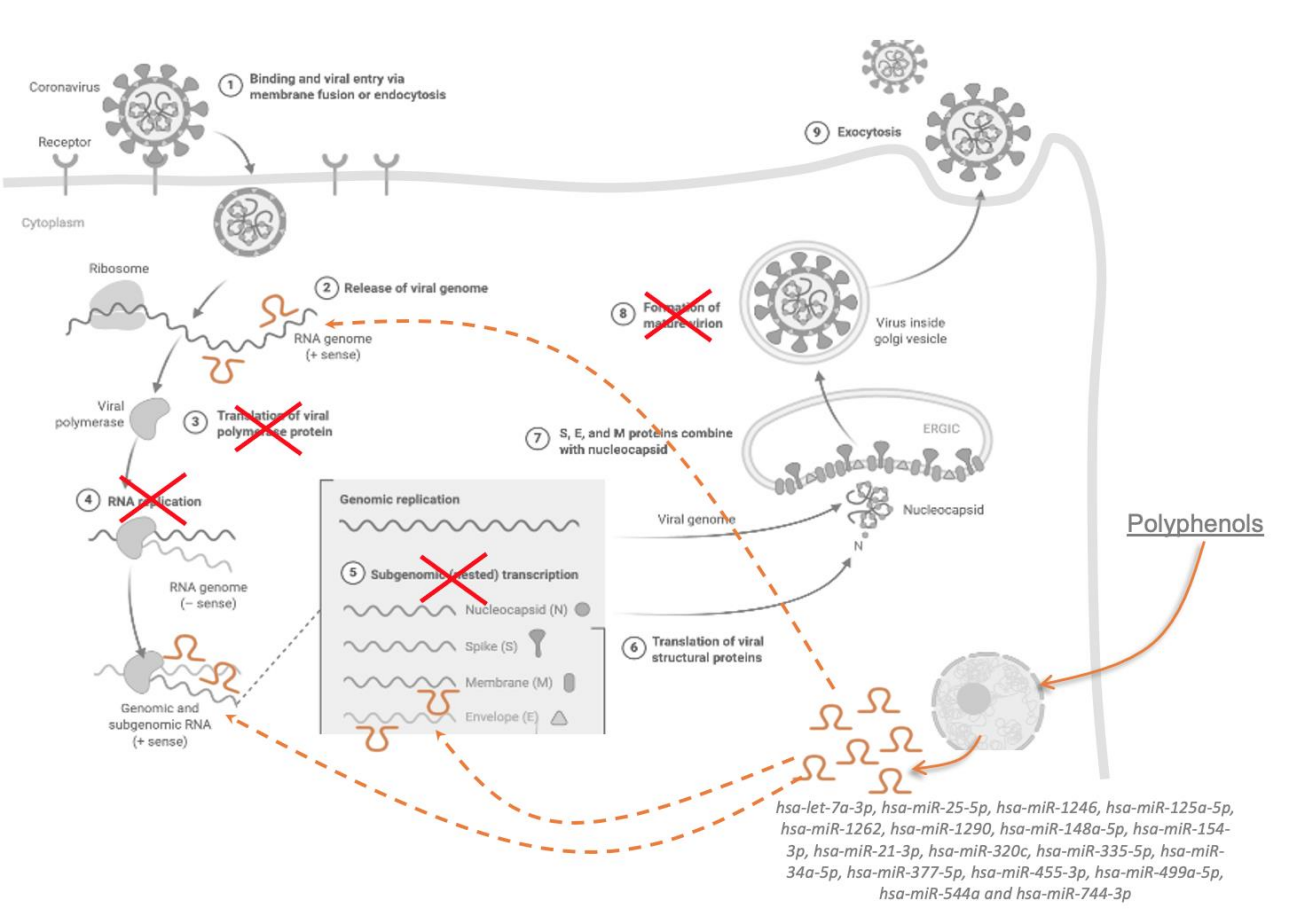
Potential mechanism of action of polyphenols on SARS-CoV-2 replication via modulating host cell miRNA expression (from Biorender.com).

## Conclusion

The role of host miRNAs in viral infection, both pro-viral and antiviral properties, have been little described, particularly for SARS-CoV-2. Nevertheless, the existing knowledge described in this paper can provide solid bases to support the hypothesis that polyphenols can have protective properties against SARS-CoV-2 by modulating the expression of miRNAs of the host cells, hypothesis that can be corroborated with few studies that suggested that these bioactives can interact with ACE2 or other proteins involved in COVID-19 (Fig. 3). It is therefore urgent to develop studies to assess their capacity to prevent COVID-19 complications, such as *in vitro* studies with alveolar and endothelial cells, animal studies with SARS-CoV-2 infection along with diets supplemented with polyphenols. It is primordially important also to evaluate correlation between intake of polyphenols by the population and the incidence of COVID-19. Such study will provide direct demonstration of the impact of polyphenols on prevention of SARS-CoV-2 infection and development of complications.
